# Mass-forming pancreatitis versus pancreatic ductal adenocarcinoma: CT and MR imaging for differentiation

**DOI:** 10.1186/s40644-020-00324-z

**Published:** 2020-07-23

**Authors:** Wolfgang Schima, Gernot Böhm, Christiane S. Rösch, Alexander Klaus, Reinhold Függer, Helmut Kopf

**Affiliations:** 1 Department of Diagnostic and Interventional Radiology, Goettlicher Heiland Krankenhaus, Barmherzige Schwestern Krankenhaus, 1170 Wien, Dornbacher Strasse 20–30, St. Josef-Krankenhaus, Vienna, Austria; 2 Department of Radiology, Ordensklinikum, Linz, Austria; 3 Department of Surgery, Ordensklinikum, Linz, Austria; 4 Department of Surgery, Barmherzige Schwestern Krankenhaus, Vienna, Austria

**Keywords:** Pancreas, Pancreatic cancer, Chronic pancreatitis, Mass-forming chronic pancreatitis paraduodenal pancreatitis, Autoimmune pancreatitis

## Abstract

Various inflammatory abnormalities of the pancreas can mimic pancreatic ductal adenocarcinoma (PDAC) at cross-sectional imaging. Misdiagnosis of PDAC at imaging may lead to unnecessary surgery. On the other hand, chronic pancreatitis (CP) bears a greater risk of developing PDAC during the course of the disease. Thus, differentiation between mass-forming chronic pancreatitis (MFCP) and PDAC is important to avoid unnecessary surgery and not to delay surgery of synchronous PDAC in CP.

Imaging features such as the morphology of the mass including displacement of calcifications, presence of duct penetrating, sign appearance of duct stricturing, presence or absence of vessel encasement, apparent diffusion coefficient (ADC) value and intravoxel incoherent motion (IVIM) at diffusion-weighted imaging (DWI), fluorodeoxyglucose (FDG) uptake in PET/CT, and mass perfusion parameters can help to differentiate between PDAC and MFCP. Correct interpretation of imaging features can appropriately guide biopsy and surgery, if necessary. This review summarizes the relevant computed tomography (CT) and magnetic resonance imaging (MRI) features that can help the radiologist to come to a confident diagnosis and to guide further management in equivocal cases.

## Introduction

The association of chronic pancreatitis (CP) with ductal adenocarcinoma of the pancreas (PDAC) is well known and has been reported in the literature [1–3]. On the other hand, CP may present as a mass-forming type, mimicking PDAC. Likewise focal autoimmune pancreatitis (AIP) and paraduodenal pancreatitis may present with imaging appearances not easily distinguishable from PDAC. So at imaging similar appearance of the two entities or a combined occurrence may cause confusion for the reader of CT or MRI studies. This confusion may lead to misdiagnoses and subsequent surgical resection of a benign inflammatory mass or, vice versa, a delay in diagnosis of potentially resectable PDAC developing in CP.

The aim of this review is to describe the CT and MR features that can help to distinguish between mass-forming chronic pancreatitis or other forms of focal pancreatitis and PDAC. The imaging diagnosis is not always straightforward, so further imaging modalities such as endosonography or invasive procedures may help to corroborate the diagnosis.

## The clinical problem of mass-forming chronic pancreatitis

CP is a disease of recurrent or ongoing, prolonged pancreatic inflammation, which is characterised by the development of irreversible morphologic and functional abnormalities. Macro-morphologic and histologic changes include fibrosis and atrophy of the gland, as well as stricturing of the pancreatic duct with ductal dilatation. Although atrophy is one of the main features of CP, occasionally mass-like focal enlargement of the pancreatic parenchyma may occur, usually in the pancreatic head. No large studies have actually described the incidence of the mass-forming type in patients with chronic pancreatitis [4, 5]. Reported incidences of up to 50% of patients may be grossly overestimated [6]. In the clinical experience of the authors probably not more than 10–20% of patients with CP develop a mass-like inflammatory lesion. However, for surgical and endoscopic treatment strategies, the pancreatic head plays a key role. Causing stenosis of the common bile duct, the pancreatic duct and the duodenum, as well as vascular encasement, the inflammatory mass is seen as the pacemaker of chronic pancreatitis [7–9]. So the vast majority of these masses develop in the pancreatic head, which is exactly the site of predominant occurrence of PDAC.

### Risk of pancreatic Cancer in chronic pancreatitis

Lowenfels et al. were the first to describe a significantly increased risk of developing pancreatic cancer in patients with CP [10]. They reported for subjects with a minimum of 2–5 years of follow-up a standardized incidence ratio of 14.4–16.5. Cumulative risk of pancreatic cancer increased over time, reaching 1.8% at 10 years and 4.0% at 20 years after the diagnosis of CP [10]. Their results were corroborated by Talamini et al., who found a standardized incidence ratio of pancreatic cancer in CP patients of 13.3, with an even higher risk in smokers [11]. A recent meta-analysis of Kirkegard et al. suggested that CP increases the risk of pancreatic cancer [1]. The association between CP and cancer diminishes with long-term follow-up: from 16 times the risk in the first 2 years after CP diagnosis to 8 times the risk after 5 years follow-up and “only” 3.5 times higher after at least 9 years follow-up. Other studies have also shown that a remote history of acute pancreatitis may precede or accelerate carcinogenesis in PDAC [12, 13]. Thus, the association of pancreatitis and pancreatic cancer development is clearly established. It is therefore of upmost importance to identify patients with suspicion of cancer early during their course of pancreatitis. In patients with autoimmune pancreatitis (AIP) or paraduodenal pancreatitis an increased risk of developing PDAC has not been reported.

### Pancreatic ductal adenocarcinoma as an incidental finding in chronic pancreatitis

The association of CP and PDAC is underlined in the study of Birgin et al. [2], which found that 30.8% of patients with CP, who underwent surgery, also had synchronous PDAC. Not surprisingly, there was more advanced lymph node involvement in PDAC patients with CP than in the control group of patients with PDAC without CP. Malinka et al. [14] elucidated in a large retrospective study the prevalence of incidental PDAC found at pancreatic resections for CP. PDAC was histopathologically found in the surgical specimen of 7.1%, with an overall survival of 11.7 months (vs. 216.1 months post surgery in CP patients without PDAC). These results underline, firstly, the importance of careful evaluation of CP patients at cross-sectional imaging to make a correct diagnosis and, secondly, the prognostic significance of early surgery in CP.

A recent study assessed the incidence of pancreatic cancer after surgery for CP. In the post-operative follow-up 1.8% of patients were diagnosed with pancreatic cancer based on histology, with a cumulative incidence of 1.48% at 3 years, 2.63% at 6 years and 3.71% at 9 years after surgery for CP [15]. It should be noted that the incidence of pancreatic cancer was significantly lower in CP patients who had received surgery for CP than in those, who had not undergone surgery [16]. These data underline the importance of cross sectional imaging in CP patients to diagnose PDAC early during development by taking subtle signs of tumour formation into account.

## Mass-forming chronic pancreatitis: imaging features

**Fig. 1:**
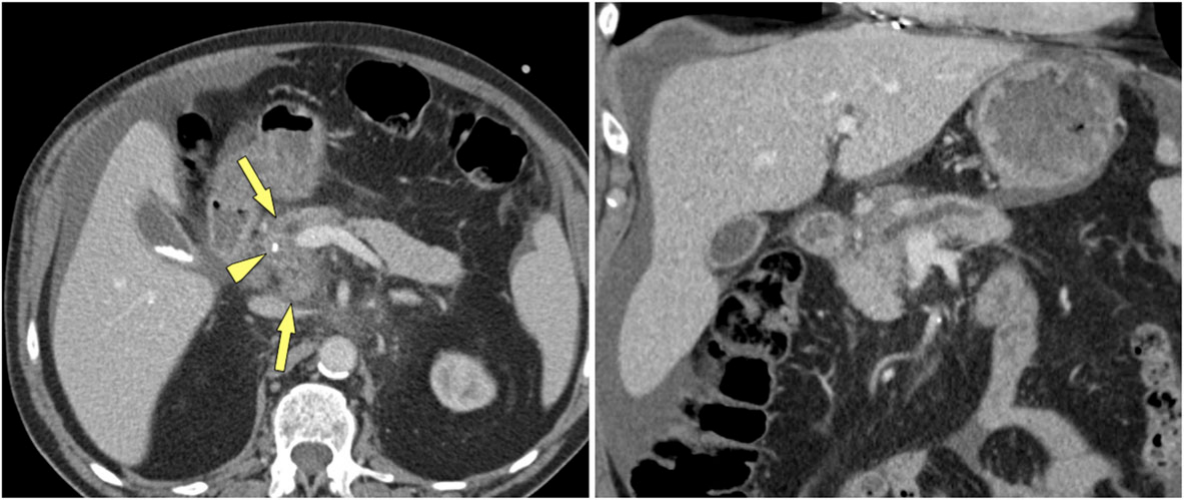
MFCP of the head in a 52 year-old male patient. **a** Axial and **b** coronal MDCT images demonstrate a hypoattuatting mass in the pancreatic head (arrows) with subsequent dilatation of the pancreatic duct. There is a single small parenchymal calcification (arrowhead), which was not intraductally located according to multiplanar imaging. No other signs of CP are present. A confident diagnosis of MFCP cannot be made

**Fig. 2:**
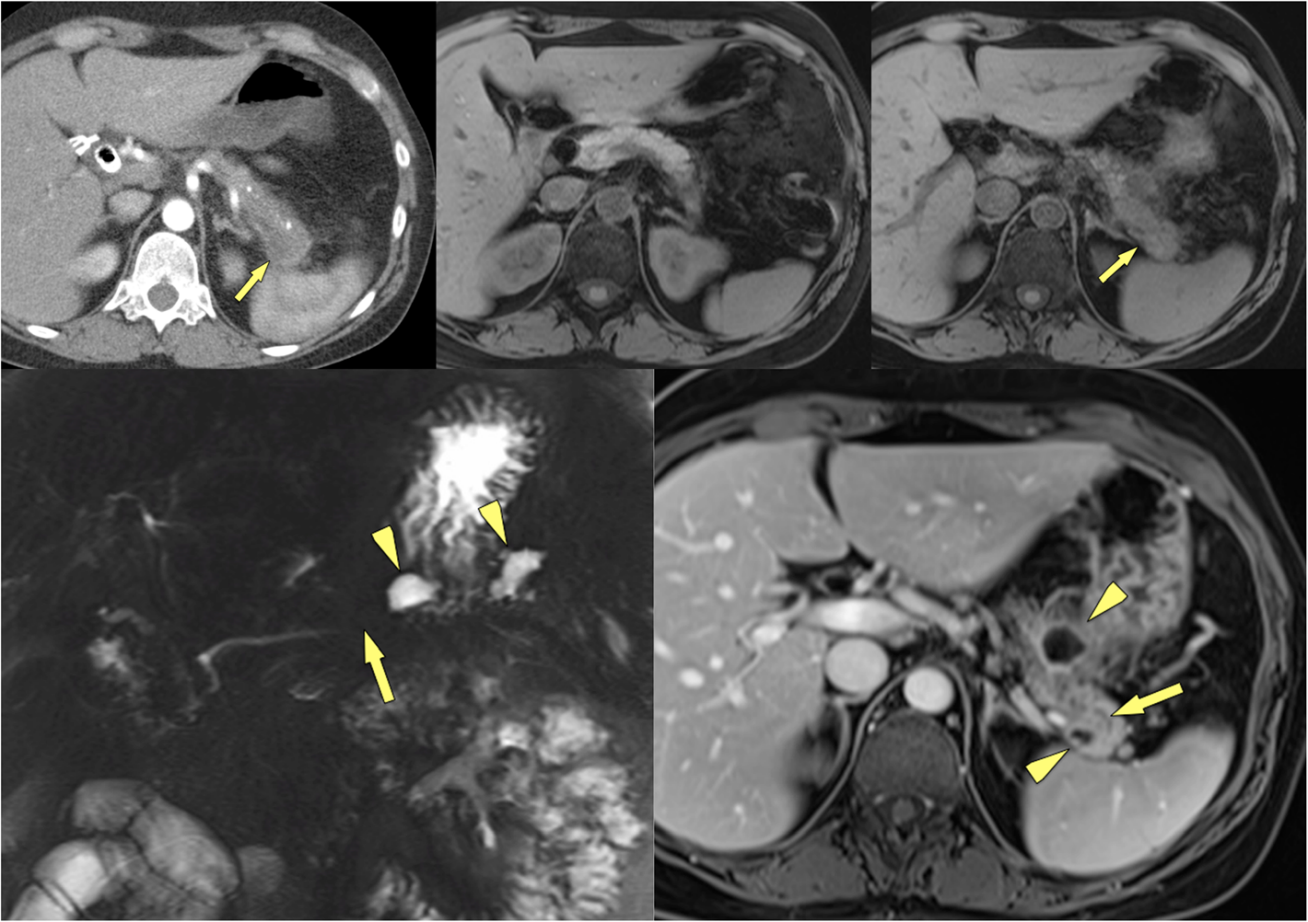
MFCP of the tail (35 y, female). **a** Incidental note of an pancreatic tail mass (arrow) is made during CT angiography. There are multiple stippled calcification in the tail. **b**, **c** T1w GRE fatsat images show normal SI of the body and markedly low SI of the tail (arrow). **d** MRCP shows a stricture at the junction of pancreatic body and tail (arrow) and two fluid collections in the tail (arrowheads). **e** Gadolinium-enhanced image shows hypovascular mass and minimal ductal dilatation in the mass (arrow). The two small fluid collections adjacent to the pancreas are pseudocysts (arrowheads). Clues to the diagnosis of MFCP are lack of a duct-obstructing mass at the stricture at the junction of body and tail, presence of pseudocysts and parenchymal calcifications (at CT)

**Fig. 3:**
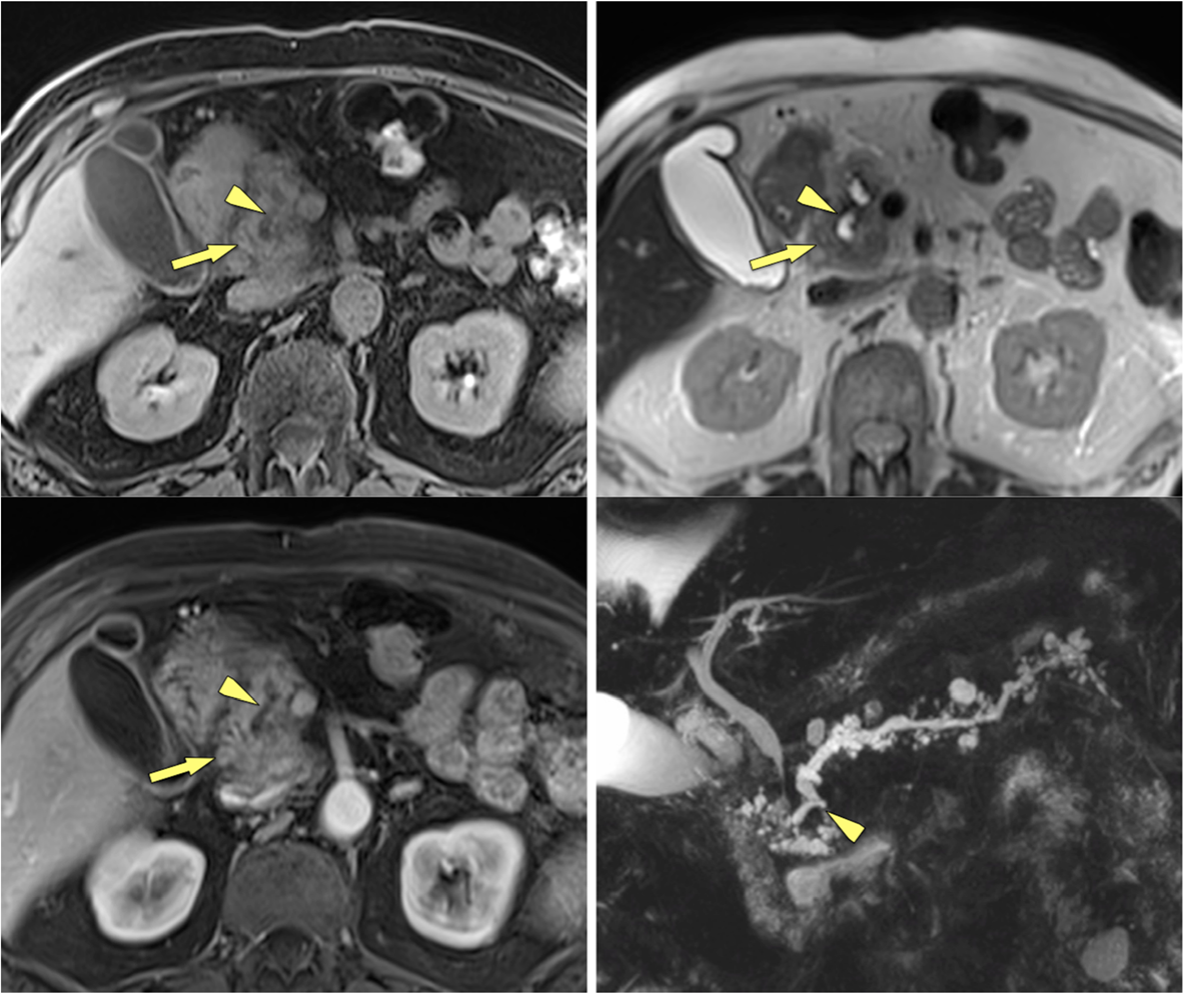
MFCP of the head (86 y, male). **a** T1w GRE fatsat, **b** T2w TSE, and **c** gadolinium-enhanced T1w GRE images show a mass in the head (arrows). The panc. duct traverses the mass (arrowheads) (duct-penetrating sign), which is typical for an inflammatory mass. **d** MRCP even better shows duct-penetrating sign (arrowhead). There are also typical signs of CP with ductal irregularities and dilated side branches throughout the gland

The imaging appearance of MFCP has been described in detail [6, 17–19]. The typical features of MFCP at MDCT are a hypoattenuating mass at unenhanced CT, which is hypovascular on contrast-enhanced scans (Fig. 1). Masses are most often located in the pancreatic head, but can also be found in the body or tail of the pancreas (Fig. 2). These masses are usually hypointense at T1-weighted gradient-recalled echo (GRE) MR imaging and iso- to hyperintense at T2-weighted MRI. MFCP is hypointense at arterial phase gadolinium-enhanced MRI, with moderate enhancement at venous-phase scans, showing moderate hypointensity to isointensity in later phases (Fig. 3). All these imaging features are very similar in MFCP and PDAC. However, there are imaging features, which help to differentiate between the two entities.

### Calcifications in chronic pancreatitis and displacement of calcifications

**Fig. 4:**
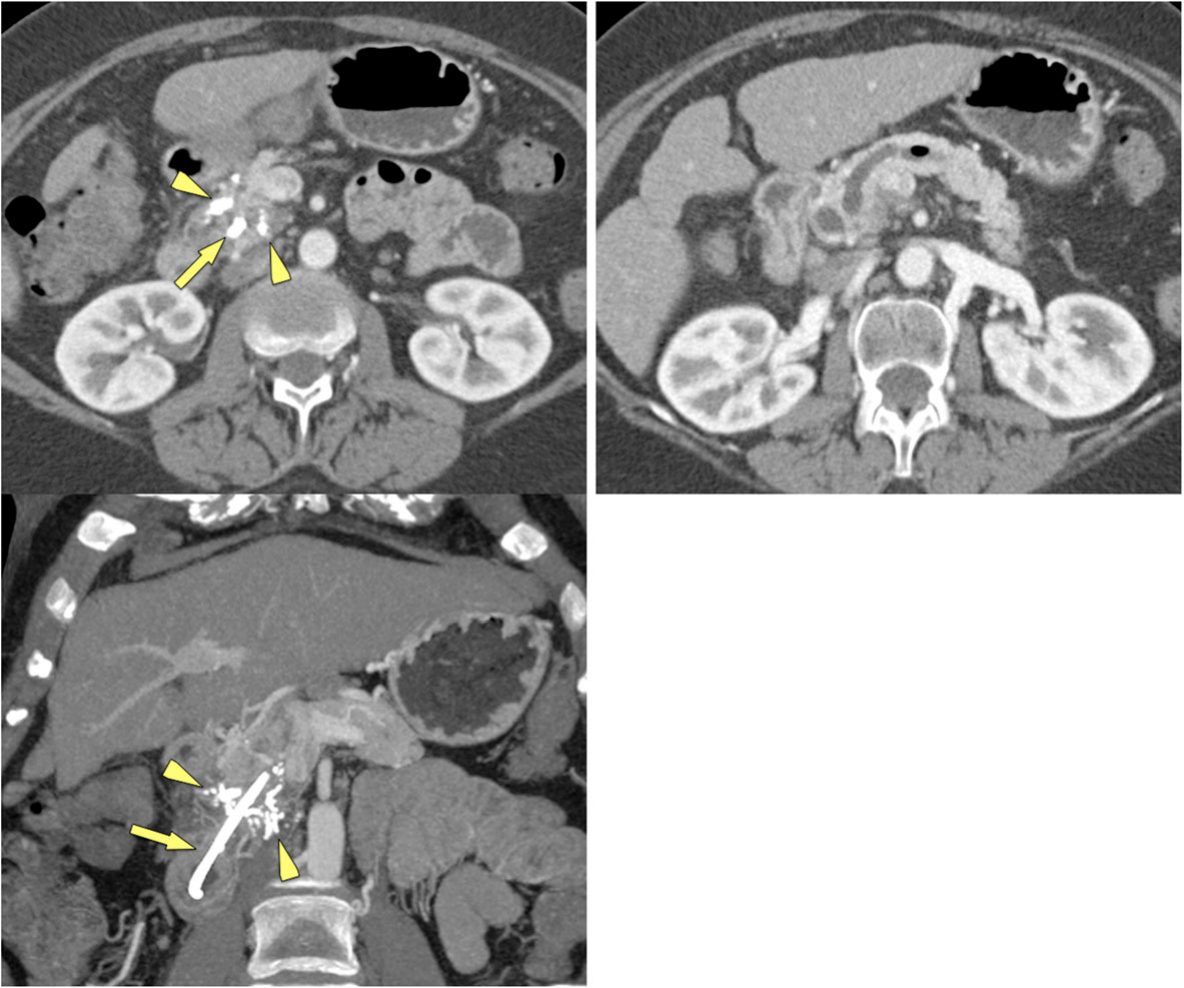
Calcified MFCP of the head (57 y, male). **a, b** Axial contrast-enhanced MDCT shows a hypoattenuating mass of the head with coarse calcifications (arrowheads) and a pancreatic plastic stent in place (arrow). The pancreatic duct is dilated. There are no signs of CP in the rest of the pancreas. **c** Paracoronal MDCT reformation demonstrates the stent (arrow) in the pancreatic head and the parenchymal calcifications (arrowheads) to better advantage

**Fig. 5:**
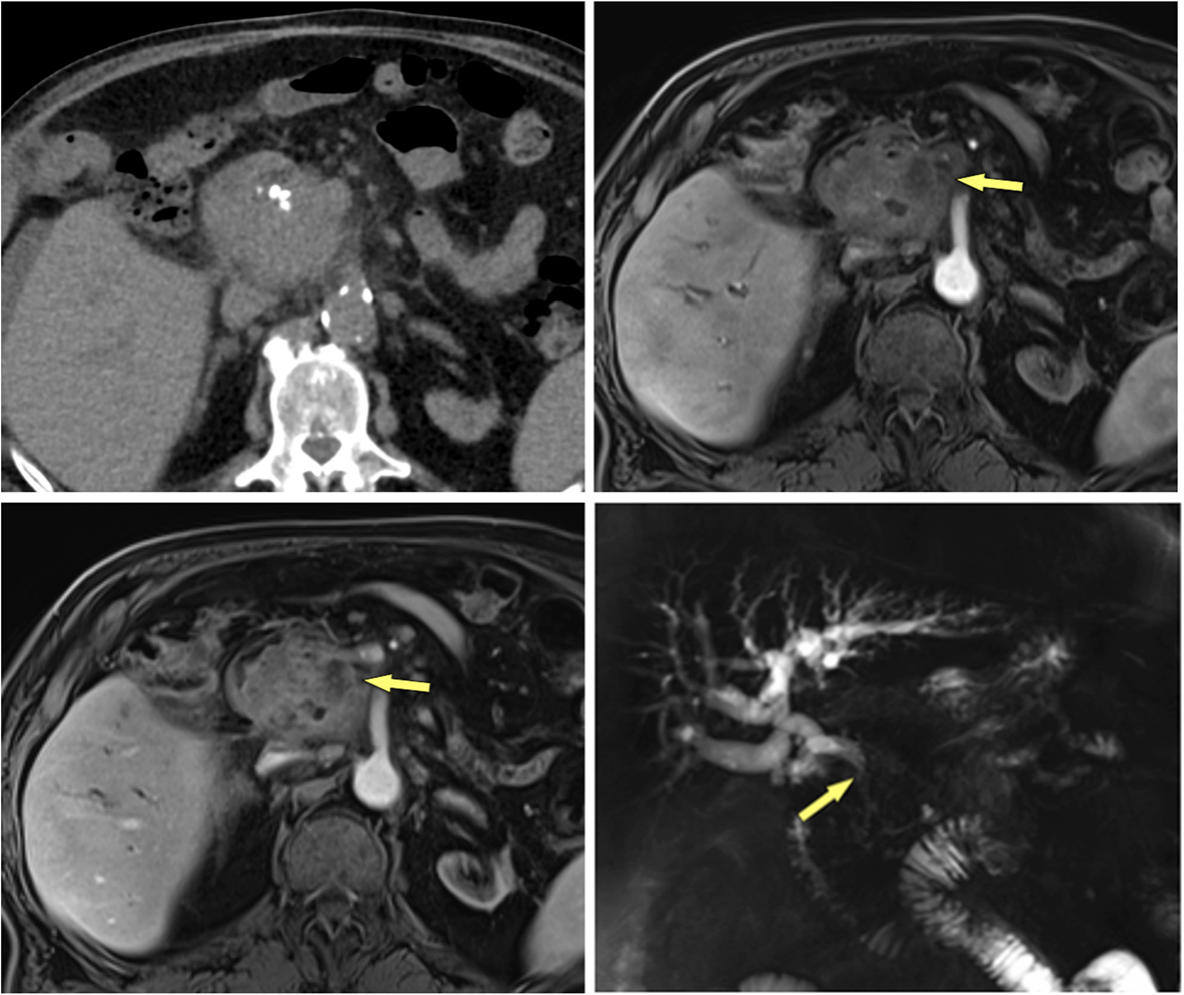
Non-calcified soft tissue mass with calcifications located in the periphery: PDAC (71 y, male). **a** Unenhanced CT shows a large soft tissue mass with marginal calcifications. **b, c** Gadolinium-enhanced T1w GRE images in the arterial and venous phases show a hypovascular mass located in the medial part of the pancreatic head (arrows). Thus the calcification seen at CT are not located in the mass, but in the adjacent parenchyma. **d** MRCP shows abrupt stenosis of the CBD (arrow) with dilatation. Surgical histology revealed PDAC in CP

**Fig. 6:**
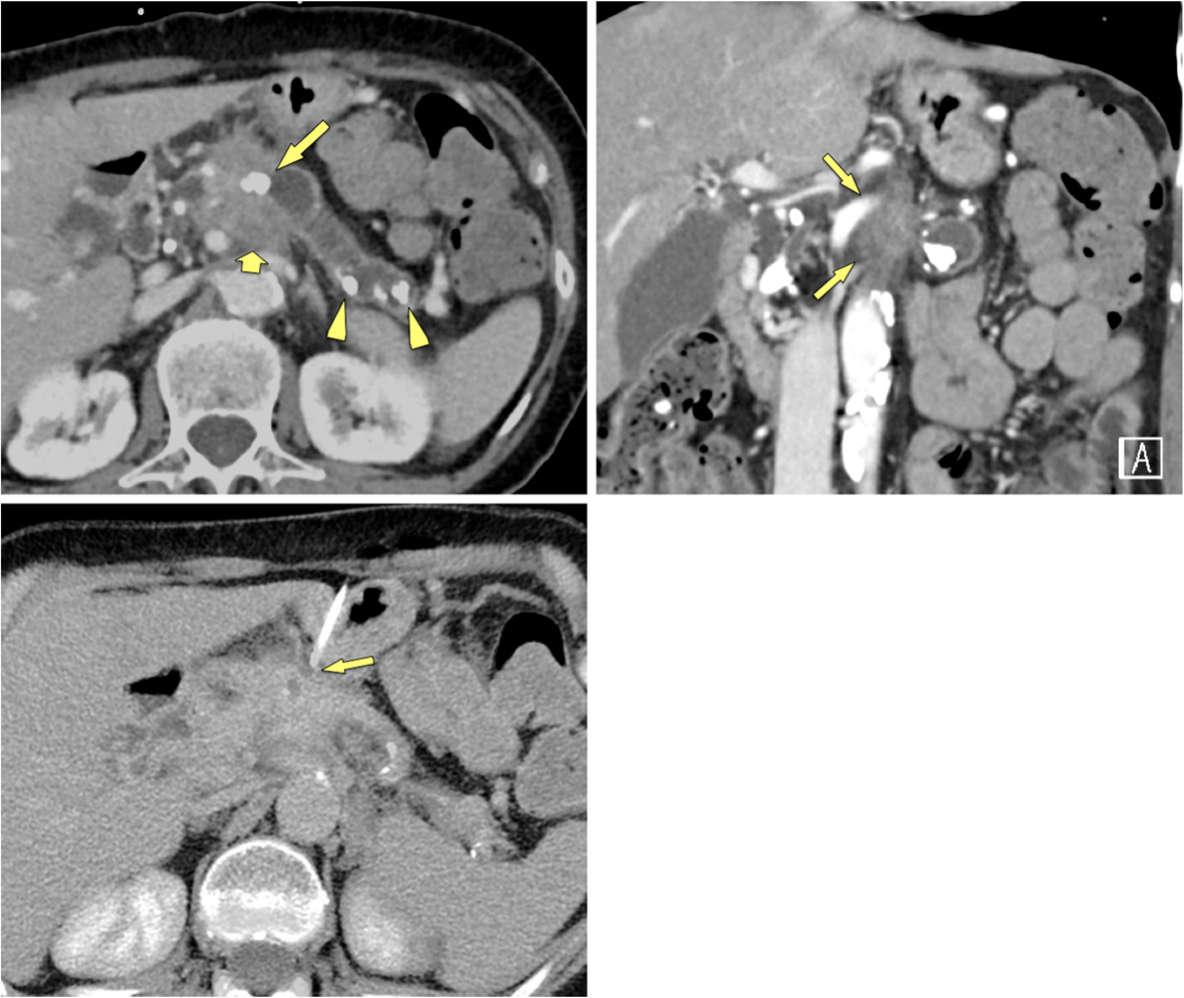
Soft tissue mass with vessel encasement in calcified CP: locally advanced PDAC (66 y, female). **a** Contrast-enhanced MDCT shows signs of severe CP with parenchymal calcifications (arrowheads) and a large intraductal stone (arrow) with duct dilatation. However, there is a non-calcified soft-tissue mass present, which infiltrates into the mesenteric root (thick arrow). **b** Coronal reformation shows encasement of the superior mesenteric artery by the mass (arrows), which is highly suspicious for PDAC. **c** Diagnosis was confirmed by percutaneous CT-guided transgastric biopsy (arrow)

Parenchymal calcifications are seen in approximately 60% of CP patients [20] (Fig. 4) and the combination of diffused parenchymal calcifications with ductal calcifications, atrophy and cystic lesions is quite specific for the diagnosis of CP [21]. The occurrence of calcifications is not pathognomonic, as neuroendocrine tumours, intraductal papillary mucinous neoplasms (IPMN) and PDAC may also occasionally show spotted calcifications. However, in these neoplasms the calcifications tend to be neither diffuse nor intraductally located. The longitudinal change of parenchymal calcifications over time with development of a new soft tissue mass displacing calcifications or the presence of a soft tissue mass in a diffusely calcified chronic pancreatitis should raise the suspicion of newly developing PDAC in CP (Figs. 5, 6).

### Duct-penetrating sign

The presence of a smooth narrowing of the pancreatic duct traversing a pancreatic mass without abrupt or complete obstruction is strongly in favour of the diagnosis of an inflammatory pseudotumour [22] (Fig. 3). The pathophysiologic basis for the duct-penetrating sign is the fact that a PDAC as a densely fibrotic tumour usually causes abrupt narrowing or even complete obstruction of the pancreatic duct, whereas an inflammatory mass most often results in gradual stenosis, with visualisation of the pancreatic duct throughout the mass. This sign, described by Ichikawa et al. in 2001, was found to be accurate in 94% of patients with MFCP or PDAC, and it is now widely used in clinical practice. Duct-penetrating sign is best seen on magnetic resonance cholangiopancreatography (MRCP), with visualisation of the duct and stenosis sometimes improved by Secretin administration [23]. With MRCP not only the main duct, but also side branches penetrating an inflammatory mass can be visualized.

### Double-duct sign

**Fig. 7:**
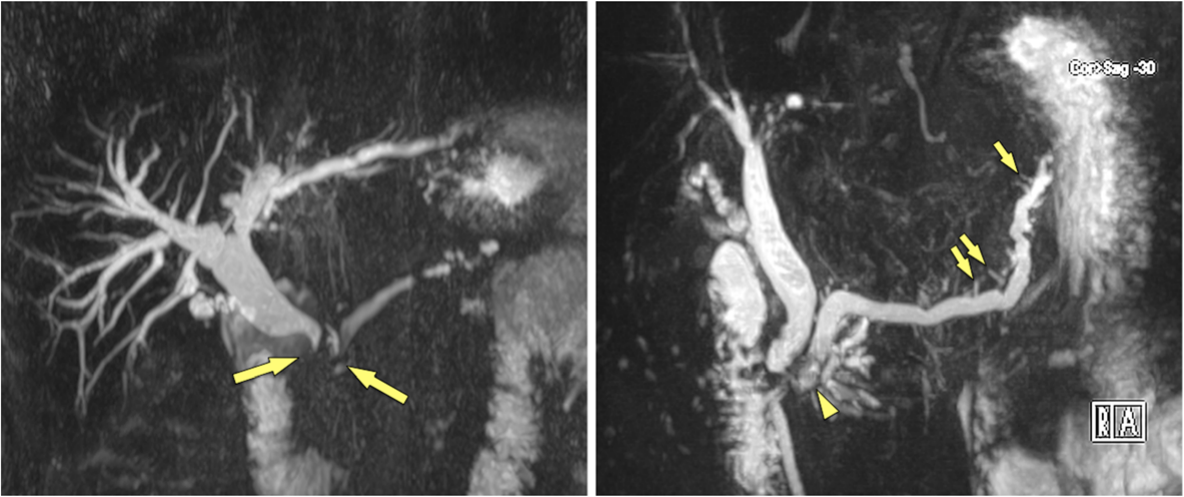
**a** Double duct sign in PDAC (62 y, male). MRCP shows abrupt cut-off of common bile duct and main pancreatic duct in the pancreatic head (arrows), very suggestive of neoplasm. Histology confirmed PDAC in this patient. **b** Lack of double duct sign in CP (57 y, female). MRCP shows ducts dilatation without abrupt cut-off, severe contour irregularities of main duct with dilated side branches (small arrows) typical for CP. There are irregular filling defects in the main duct, suggestive of stones (arrowhead)

**Fig. 8:**
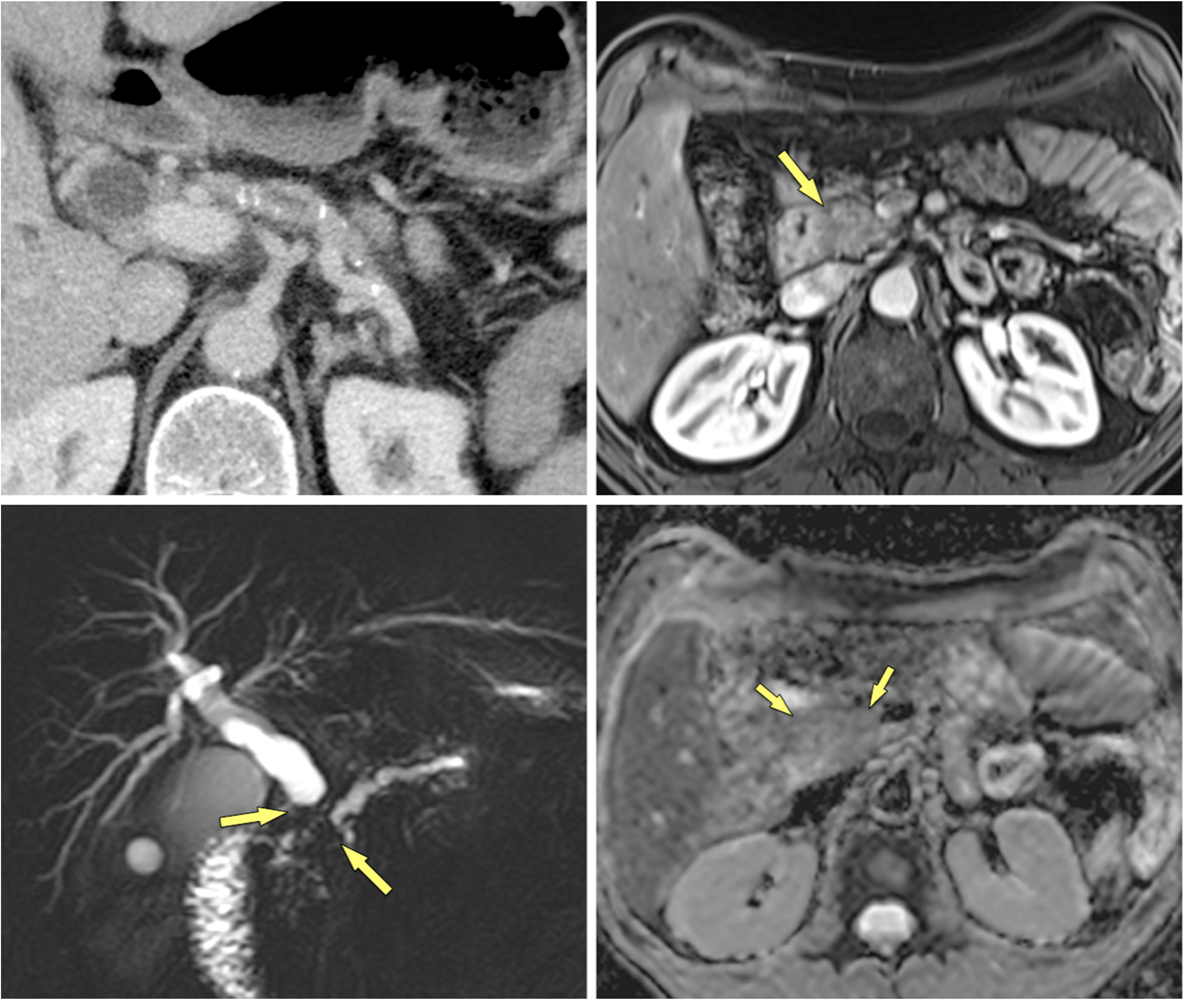
“False-positive” double duct sign in a patient with MFCP (67 y, female). **a** Axial MDCT shows severely atrophic pancreas with stippled calcifications in long-standing CP. **b** In the head there is a hypovascular mass, which causes **c** abrupt cut-off (arows) of common bile duct and main pancreatic duct (double duct sign), highly suspicious for PDAC developing in CP. **d** ADC shows only minimally restricted diffusion of the mass (arrows) (ADC value 1.51 × 10^− 3^ mm^2^/s), which is more in favour of inflammation. Patient underwent surgery, which revealed an inflammatory mass and no cancer

The common bile duct (CBD) and the main pancreatic duct form a junction at a level of the major papilla. Thus, an obstruction at the papilla or in the periampullary region (in the pancreatic head) may cause stenosis of both ductal systems with subsequent pre-stenotic ductal dilatation. This imaging feature of dilatation of both ductal systems is known as double-duct sign (Fig. 7). It is much more often seen in PDAC than in an inflammatory condition, with a reported incidence of up to 80%. The presence of a double-duct sign by itself is not pathognomonic for PDAC. In cancer-induced stenosis usually there is abrupt cut-off of the CBD, with similarly abrupt stenosis of the main pancreatic duct at a corresponding level. Double-duct sign may occur in MFCP or autoimmune pancreatitis, but is usually defined by a more smooth and tapered narrowing of the CBD and main pancreatic duct with less severe prestenotic dilatation. However, in rare cases MFCP may cause a double duct sign indistinguishable from PDAC (Fig. 8), with progressive biliary dilatation (and cholestasis) as well as pain. In these cases surgery should be considered early.

### Morphology of pancreatic duct and side branches

The pancreatic duct is usually visible at CT or MRI from the papilla to the tail. The diameter of the duct should not exceed 3 mm in the pancreatic head and 2 mm in the body and tail [24, 25]. A duct diameter of more than 3.5 mm in the head is definitely considered abnormal. Ductal dilatation is defined as a diameter exceeding the upper limit of normal or, in case of a stenosis, an abrupt calibre change upstream to the stenosis, even if the duct diameter does not exceed the upper normal limit. Careful assessment of the pancreatic duct calibre upstream and downstream of a stenosis is sought. Curved-planar reformations along the pancreatic duct may help in this respect.

Morphology of pancreatic duct dilatation due to PDAC or an inflammatory stricture in CP may differ significantly. In case of advanced stage CP not only focal strictures, but also contour irregularities of the duct upstream are seen. Likewise, in CP usually dilated and deformed side branches are visualized, which are called a “chain of lakes” at MRCP. Pancreatic duct dilatation upstream to PDAC is usually severe, but quite smooth and also includes severe parenchymal atrophy.

### Vessel encasement

Extension of a pancreatic mass towards the celiac trunk or the superior mesenteric artery with soft-tissue density encasement of the vessels is highly suggestive of PDAC, although it can rarely be observed in patients with chronic inflammation. Venous deformation of the confluence, superior mesenteric vein or splenic vein is more likely in PDAC than in MFCP, but its occurrence is not at all specific. Especially occlusion of the splenic vein with extensive collateral formation is a typical feature in patients with recurrent pancreatitis of the body and tail. Thus, only the image feature of soft-tissue encasement of the peripancreatic arteries is quite specific, but it indicates presence of already locally advanced (and most likely unresectable) PDAC (Fig. 6).

### Perfusion-CT

Recent studies on perfusion-CT revealed promising results regarding differentiation between MFCP and PDAC. Perfusion-CT not only allowed reliable differentiation between normal parenchyma and MFCP and PDAC, but also between the latter two [26, 27]. The mean blood flow (BF), blood volume (BV) and permeability surface area product (PS) were significantly higher in MFCP than in adenocarcinoma. There was no significant difference in mean transit time (MTT) between MFCP and adenocarcinoma [26]. The combination of threshold values for BV, BF and PS yielded excellent sensitivity and specificity for differentiation between MFCP and PDAC [27]. However, these results have to be corroborated by other studies with perfusion software of different vendors.

### Perfusion and diffusion-weighted MRI

A preliminary study evaluated the role of gadolinium-enhanced perfusion-MRI for differentiation between MFCP and PDAC. Although it showed differences in perfusion curves between MFCP and PDAC, this feature alone did not allow reliable diagnosis [28]. The combination of perfusion MRI and DWI achieved better results. The mean ADC value of PDAC was significantly lower than that of MFCP (1.17 ± 0.23 < vs. 1.47 ± 0.18, *p* < 0.01, obtained at 3.0 T MRI). Recently intravoxel incoherent motion (IVIM) DWI has been shown to be able to differentiate between PDAC and AIP and between PDAC and CP [29–31]. The slow component of diffusion D_slow_ and the perfusion fraction f were significantly higher in CP than in PDAC [30].

### Multimodality imaging features and biopsy

There is no single imaging feature that allows reliable differentiation between MFCP and PDAC or even PDAC developing CP. It is a combination of features, which are either present or absent, which may drive you towards one or the other diagnosis. MFCP patients likely show diffuse spotted or coarse parenchymal calcifications and/or ductal calcifications with duct-penetrating sign. ADC values in MFCP are higher than in PDAC (Fig. 8d). Pseudocyst formation and thickening on the right renal fascia are very common in MFCP [32]. At FDG-PET/CT the SUV of MFCP is usually lower (cut-off value 4.90) than in PDAC [32]. At contrast-enhanced ultrasound perfusion parameters differ significantly between MFCP and PDAC, with MFCP displaying shorter contrast agent arrival time and shorter time-to-peak [33].

**Fig. 9:**
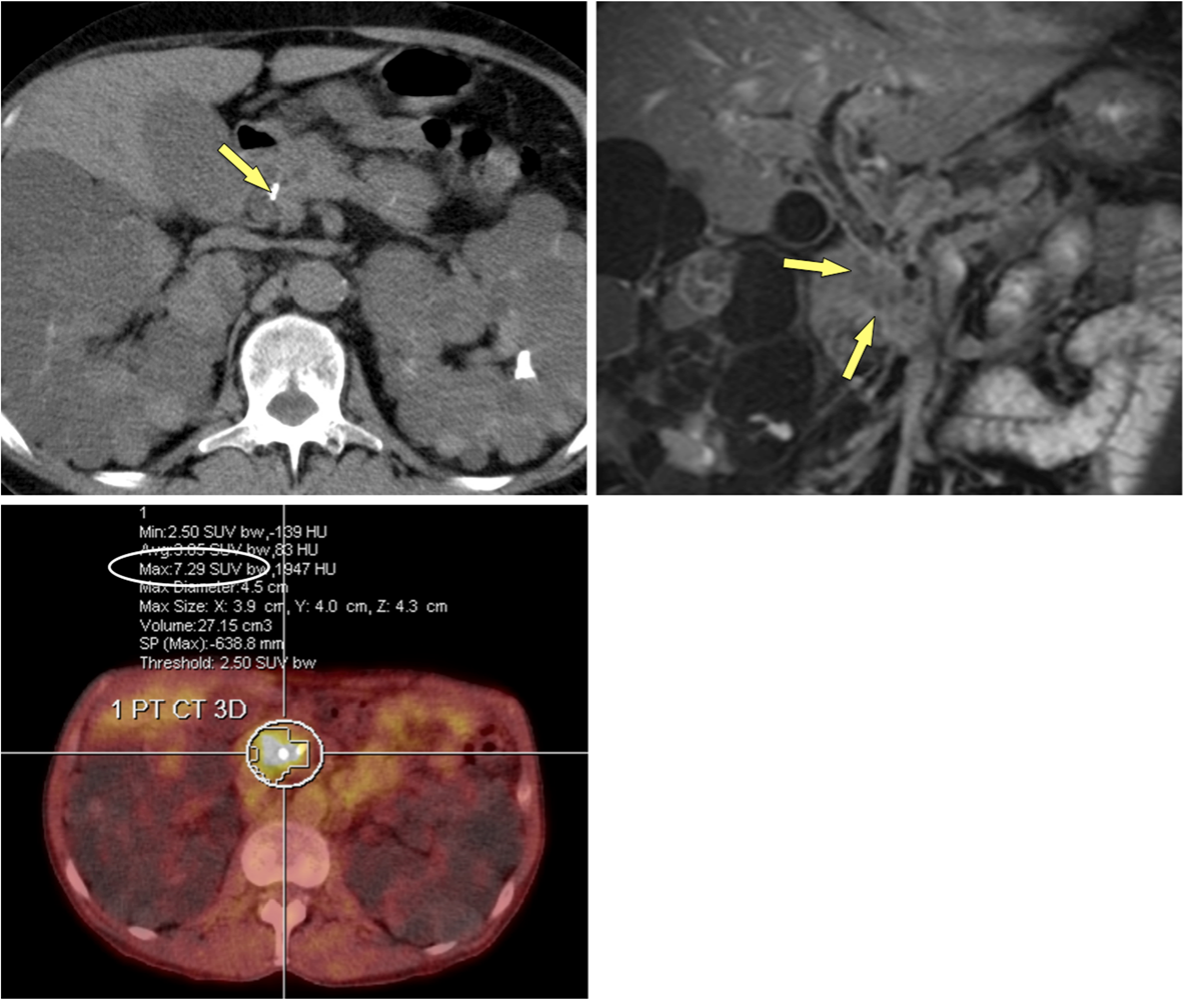
CP and polycystic kidney disease (55 y, male): value of FDG-PET/CT for tumour detection. **a** Unenhanced CT shows calcification in the pancreatic head (arrow) and massively enlarged polycystic kidneys. **b** Gadolinium-enhanced T1w GRE fatsat image shows a hypovascular mass in the head (arrows), **c** which is quite hypermetabolic at FDG-PET/CT (SUV 7.29), highly suspicious for cancer. Histology revealed locally advanced PDAC stage pT3 N1 with lymphangiosis and perineural spreading

**Fig. 10:**
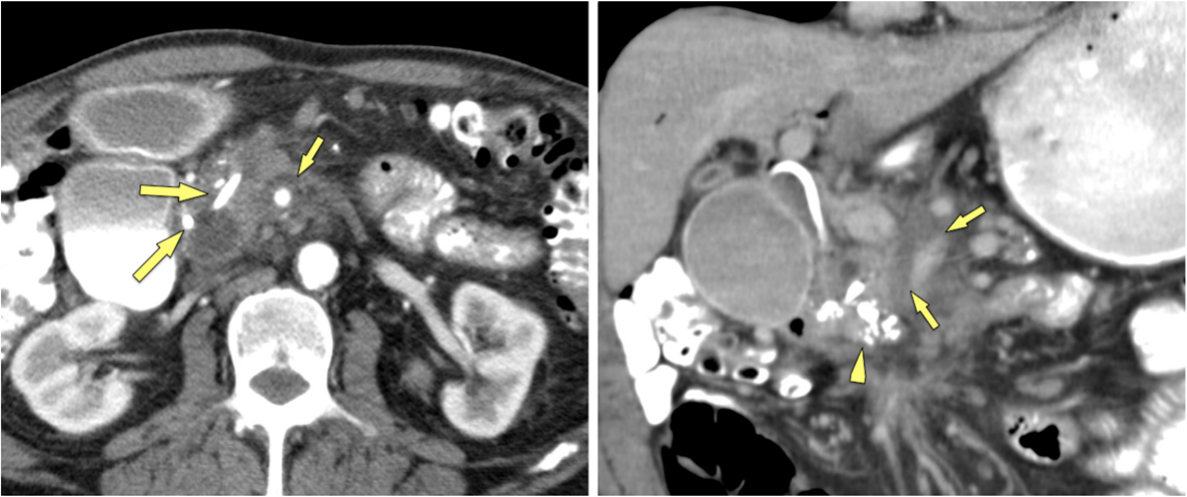
Calcific CP of the pancreatic head (70 y, male): biliary and pancreatic stents in situ. **a** Axial MDCT shows 2 stents in place (large arrows), which impairs assessment of the pancreatic head. No real mass is seen, but the fat around the superior mesenteric artery (SMA) is blurred (small arrows). **b** Coronal MDCT reformation confirms infiltration of the perivascular fat around the SMA (small arrows). Histopathology revealed PDAC. Calcifications are seen in the pancreatic head (arrowhead). Dense residual contrast is present in the duodenum and stomach after ERCP: delayed emptying is due to stenosis of the duodenum in CP

Vice versa, complete obstruction of the pancreatic duct by the mass, double-duct sign due to periampullary obstruction, arterial encasement by a soft-tissue mass, and high FDG uptake in PET/CT are much more likely in PDAC (Fig. 9). The displacement of previously seen calcifications by a newly appearing soft-tissue mass is highly suspicious of PDAC developing in CP. The presence of biliary and/or pancreatic stents may impair assessment of the pancreatic head (Fig. 10). Persistent pain despite stent treatment should raise suspicion and prompt further diagnostic procedures. Endoscopic ultrasound (EUS) and EUS-guided biopsy are in general very sensitive tools in focal masses of the pancreas. However, several studies have shown that in patients with chronic pancreatitis the sensitivity of EUS-guided biopsy drops significantly to only 50–75% [18, 34]. If you know about this limitation of EUS-guided fine-needle biopsy in CP patients, surgery has to be considered in patients with suspicious clinical findings, but equivocal clinical imaging features and negative histology. Percutaneous US-guided fine needle aspiration biopsy and core biopsy are excellent tools for diagnosis of PDAC [35, 36]. However, in the clinical scenario of differential diagnosis between PDAC and MFCP a negative result (i. e., negative for cancer) will raise questions about a sampling error.

## Focal autoimmune pancreatitis

**Fig. 11:**
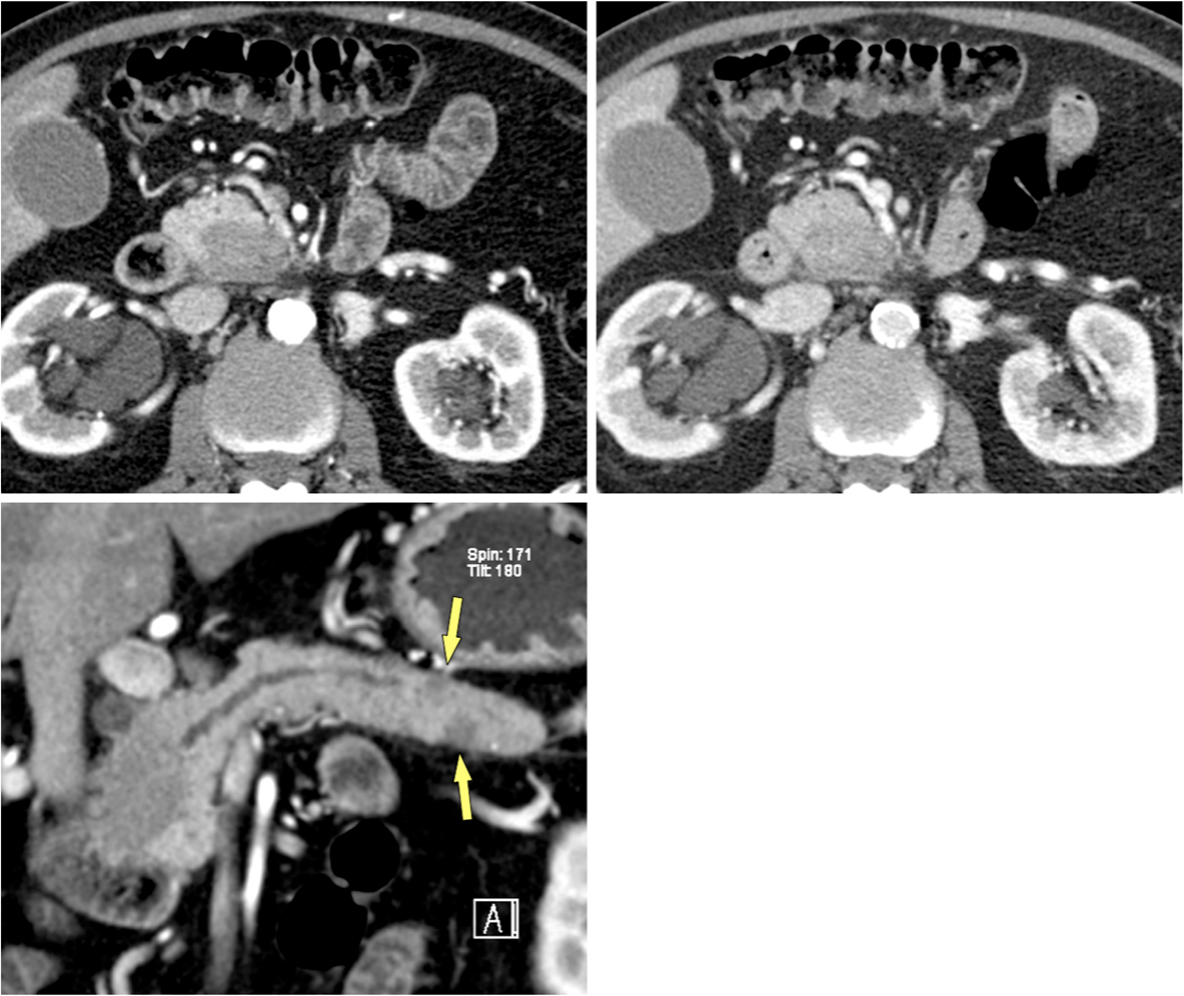
Multifocal autoimmune pancreatitis (AIP) (71 y, male). Contrast-enhanced MDCT **a** arterial and **b** portal-venous phases shows a hypoattenuating mass in the head and uncinate process with progressive enhancement in the portal-venous phase. MFCP or PDAC have to be considered in the differential diagnosis. **c** Curved-planar reformation shows not only the mass in the head, which obstructs the panc. duct, but also two smaller lesions in the tail (arrows). There are no signs of CP elsewhere. Multifocality is a clue to the diagnosis of AIP.

Autoimmune pancreatitis (AIP) is a rare form of chronic pancreatitis, which is characterized by lymphoplasmacytic infiltration with interstitial fibrosis, elevated serum IgG4 levels and a marked clinical response to corticosteroid therapy [37, 38]. AIP may present as with focal, multifocal, or diffuse infiltration of the gland (Fig. 11). The focal mass-forming type of AIP is present in 31–50% [39, 40]. Focal AIP can mimic PDAC, resulting in unnecessary pancreatic surgery for suspected cancer [41]. Several clinical and imaging features can help to differentiate focal AIP and PDAC. Focal AIP usually shows homogenous enhancement on portal-venous phase CT, a smooth narrowing of the pancreatic duct at the site of the mass (no abrupt cut-off) with only mild main pancreatic duct dilatation (≤ 5 mm) upstream, and the absence of proximal pancreatic atrophy [41]. At MRI, the mass is markedly hypointense on T1w images with lesion contrast being highest. In the enhanced phase there is decline of lesion-parenchymal contrast during the arterial phase and portal-venous phases due to progressive contrast material uptake into the mass [39]. In addition, ADC value is significantly lower in mass-forming focal AIP than in PDAC (0.96 ± 0.14 vs. 1.13 ± 0.23 × 10^− 3^ mm^2^/s at 3.0 T) [38]. The presence of typical extrapancreatic AIP manifestations (in IgG4 disease), such as features of cholangitis with duct thickening, renal involvement with bilateral patchy lesions, involvement of lymph nodes or parotid glands, may aid making a correct diagnosis [40]. If at least 4 out of 7 findings (1. early homogenous good enhancement, 2. delayed homogenous good enhancement, 3. hypoattenuating capsule-like rim, 4. absence of upstream pancreatic atrophy, 5. presence of duct-penetrating sign, 6. upstream pancreatic duct dilatation ≤4 mm, and 7. decreased ADC value) were present at MRI, then the diagnosis of mass-forming AIP could be made, for a sensitivity of 100% and a specificity of 98% [42].

## Paraduodenal pancreatitis

**Fig. 12:**
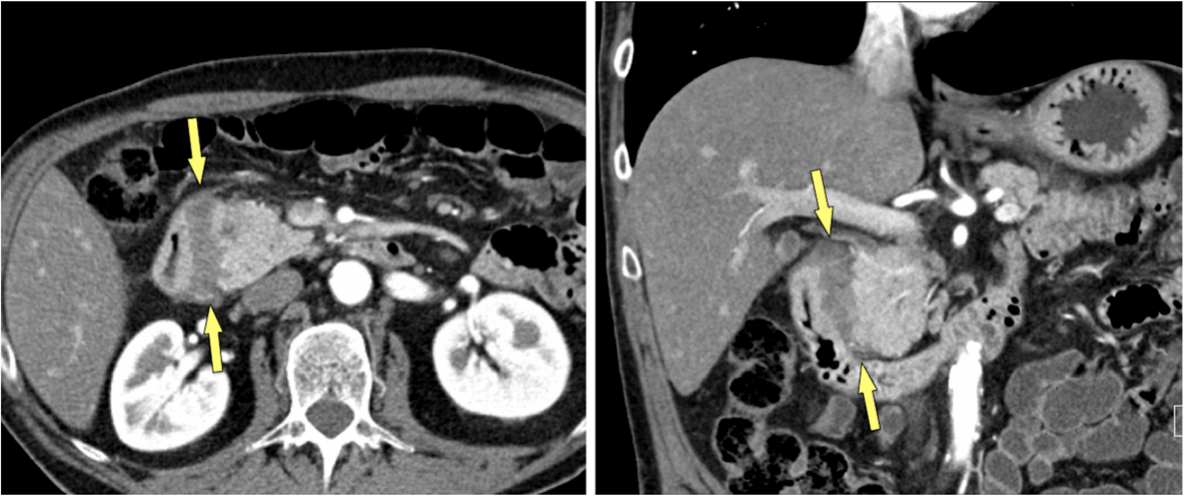
Paraduodenal pancreatitis: groove-predominant type (48 y, male). **a** Axial and **b** coronal MDCT show a hypoattenuatting mass in the groove between pancreatic head and duodenum (arrows). The pancreatic head appears normal

**Fig. 13:**
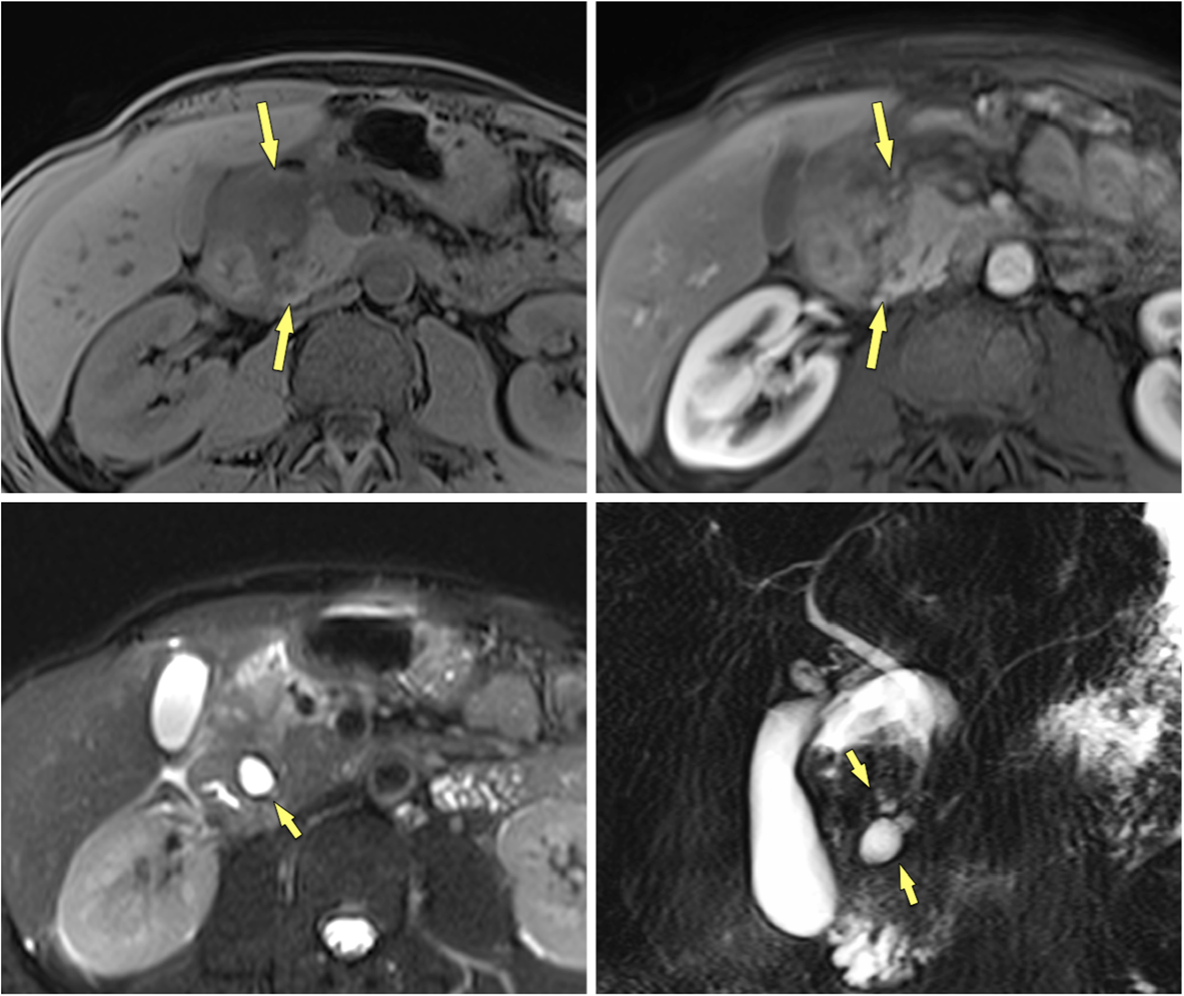
Paraduodenal pancreatitis: pancreas-involving or segmental type (44 y, male). **a** Unenhanced and **b** gadolinium-enhanced T1w GRE images show a hypointense mass (large arrows), which is hypovascular. The center of the mass is in the groove, but it also involves the pancreatic head. **c** Axial T2w TSE and **d** MRCP images shows small cysts (small arrows) in the mass, which are pathognomonic

Paraduodenal pancreatitis (formally called “groove pancreatitis”) is a distinct form of chronic pancreatitis, involving either the groove between pancreatic head and duodenum (groove-predominant type) or the groove plus the pancreatic head (pancreas-involving type) (Figs. 12, 13) [43]. The latter type is often misdiagnosed as cancer, although it lacks parenchymal atrophy of the body [43]. Histologically and at imaging a solid variant (in 32%) can be distinguished from a cystic variant (in 68%), which is a helpful clue in the diagnosis [44]. The cysts may be tiny or large and predominantly located in the groove or in the duodenal wall, with MRCP being most helpful for making the observation (Fig. 13) [45]. Pancreatic duct dilatation is most often less severe than in PDAC. PDAC arising in the periampullary region with infiltration into the duodenum can mimic paraduodenal pancreatitis. However, PDAC will then usually encase the gastroduodenal artery and cause biliary duct dilatation and overt jaundice, which are rare features in paraduodenal pancreatitis [46].

## Conclusion

In conclusion, the diagnosis of MFCP remains a challenge, although several imaging features obtained at multi-modality imaging will allow a reliable diagnosis in the majority of patients, which can be corroborated by guided biopsy. Increased risk of developing PDAC in patients with CP has to be kept in mind in order not to miss early clinical and imaging warning signs. The two special forms of mass-forming AIP and paradudenal pancreatitis need increasing awareness by the reporting radiologist, because its confident diagnosis will help to avoid unnecessary surgery.

## Data Availability

not applicable.

## Abbreviations

ADC: Apparent diffusion coefficient
AIP: Autoimmune pancreatitis
BF: Blood flow
BV: Blood volume
CP: Chronic pancreatitis
CT: Computed tomography
D_slow_: Slow component of diffusion
EUS: Endoscopic ultrasound
FDG: Fluorodeoxyglucose
IPMN: Intraductal papillary mucinous neoplasm
MFCP: Mass-forming chronic pancreatitis
MRCP: Magnetic resonance cholangiopancreatography
MRI: Magnetic resonance imaging
MTT: Mean transit time
PDAC: Pancreatic ductal adenocarcinoma
PET: Positron emission tomography
PET/CT: Positron emission tomography-computed tomography
PS: Permeability surface area product
SUV: Standard uptake value
